# Outcome-Driven Cluster Analysis with Application to Microarray Data

**DOI:** 10.1371/journal.pone.0141874

**Published:** 2015-11-12

**Authors:** Jessie J. Hsu, Dianne M. Finkelstein, David A. Schoenfeld

**Affiliations:** 1 Massachusetts General Hospital Biostatistics Center, Boston, MA, United States of America; 2 Biostatistics Department, Harvard School of Public Health, Boston, MA, United States of America; 3 Genentech, Inc., South San Francisco, CA, United States of America; University of Louisville, UNITED STATES

## Abstract

One goal of cluster analysis is to sort characteristics into groups (clusters) so that those in the same group are more highly correlated to each other than they are to those in other groups. An example is the search for groups of genes whose expression of RNA is correlated in a population of patients. These genes would be of greater interest if their common level of RNA expression were additionally predictive of the clinical outcome. This issue arose in the context of a study of trauma patients on whom RNA samples were available. The question of interest was whether there were groups of genes that were behaving similarly, and whether each gene in the cluster would have a similar effect on who would recover. For this, we develop an algorithm to simultaneously assign characteristics (genes) into groups of highly correlated genes that have the same effect on the outcome (recovery). We propose a random effects model where the genes within each group (cluster) equal the sum of a random effect, specific to the observation and cluster, and an independent error term. The outcome variable is a linear combination of the random effects of each cluster. To fit the model, we implement a Markov chain Monte Carlo algorithm based on the likelihood of the observed data. We evaluate the effect of including outcome in the model through simulation studies and describe a strategy for prediction. These methods are applied to trauma data from the Inflammation and Host Response to Injury research program, revealing a clustering of the genes that are informed by the recovery outcome.

## Introduction

Cluster analysis has been used in diverse fields to assign characteristics of an observation (or observations) into groups (clusters) so that those in the same group are more similar to each other than they are to those in other groups. In *machine learning* nomenclature, cluster analysis is described as *unsupervised learning* because there is not an outcome that informs the algorithm. It is juxtaposed to *supervised learning* that includes techniques such as discriminant analysis that are designed to use the characteristics of an observation in order to predict an outcome that is associated with it. A great deal of new interest in machine learning comes from its application to genomics and proteomics, exciting new avenues of biological research. In genomics, each observation comes from a sample of living tissue, and each characteristic is a number that quantifies the extent that a particular gene is active at the time the tissue was sampled, in the sense that RNA and eventually proteins are being produced. In proteomics, the characteristic is the amount of a specific protein in the sample. Biologists use cluster analysis to try to understand the relationship between different genes or proteins, to discover the common functionality of genes, and as a way to reduce the dimension of the data vector in preparation for other analysis.

In this paper we develop a model-based supervised clustering algorithm where the clustering of variables is informed by an outcome. The model was motivated by our work in genomics. Each observation was a sample of RNA from the white blood cells of a different patient who had recently suffered from severe blunt trauma (largely automobile crashes). The outcome was the maximum multiple organ failure score, which measures the extent of organ failure subsequent to the injury and is predictive of eventual metabolic recovery. The rest of this paper will refer to the variables as genes and the observations as samples. Our primary goal is to reduce a microarray dataset into clusters of genes that are biologically meaningful. We would like to find clusters of genes that are both correlated with each other as well as associated with patient outcome, and we hypothesize that using outcome information to drive the pattern discovery can potentially result in gene clusters that are more coherent and biologically meaningful.

The data in our example is from the Inflammation and Host Response to Injury research program, also known as the Glue Grant (http://www.gluegrant.org). The Glue Grant is a large-scale interdisciplinary study of inflammation following severe trauma or burn injury. The immune system reacts to injury by activating the inflammation response in an attempt to prevent further damage to the body. For some patients, this results in a cascade of organ failure and ultimately death, but others experience a gradual recovery and stabilization of metabolic function. It is hypothesized that the chain of events that takes place as the body tries to stabilize and recover is reflected in differential gene expression. The general aims of the Glue Grant are to uncover the biological reasons why patients have such varying responses following their injury, to understand the genomic and proteomic markers that predict clinical outcomes, and to determine the relationship between changes in gene expression and clinical features [1]. For this paper, we focus on the association between patterns in differential gene expression and metabolic recovery in patients with severe trauma.

Many methods have been developed for relating gene expression to clinical outcomes, most of which involve reducing the dimensionality of the gene expression data. One way to approach this is to identify a subset of genes that are predictive markers of outcome via univariate variable selection, stepwise selection, or constrained regression [2]. Dimension reduction can also be accomplished by principal components regression, an unsupervised procedure that reduces the gene expression values down to their principal components and incorporates the first few components that explain the majority of the predictor variation into a regression model [3]. A supervised version of this approach is partial least squares regression (PLSR) [4]. Here, both the predictors and outcome are decomposed such that the latent vectors used in the decomposition maximize their covariance.

Clustering is a widely used form of microarray dimension reduction that is based on the assumption that groups of genes are more similar to each other than others for reasons such as related functionality, shared biological pathways, or a similar effect on outcome. One approach, though computationally burdensome, is to perform a stochastic search across the entire space of possible partitions and select the optimal clustering to be the one with the highest likelihood. Another approach is to cluster the genes across patient samples via a technique such as K-means and then use the cluster expression averages in a regression model [5]. K-means clustering is a classic clustering algorithm that finds the partition of K sets that minimizes the distance of each observation to its center where each cluster center is the mean of the observations in that cluster [6]. Achieving the optimal clustering using K-means with a Euclidean distance metric is equivalent to maximizing the likelihood that corresponds to modeling gene expression as a normally distributed cluster specific fixed effect. The maximum likelihood occurs when each gene is assigned to its nearest cluster center such that the within cluster sum of squares is minimized. This approach operates under the assumption that all the genes to be clustered are independent. This is appropriate for clustering independent individuals but is flawed for clustering features that have a correlation structure (such as genes within the same patient). Rather, it is more reasonable to state that genes in the same cluster are correlated while genes across different clusters are independent. Furthermore, K-means assumes that there is only one correct clustering pattern and does not provide a measure of uncertainty associated with the cluster assignments.

A related formulation of the clustering problem is the normal mixture model, where each observation is viewed as arising from a mixture of distributions. The papers [7] and [8] discussed model-based clustering where the gene expression data is modeled as a normal mixture and clusters are determined by the Expectation-Maximization (EM) algorithm. A Bayesian approach can also be used to fit the mixture model [9]. In these approaches, the probability distribution of each gene is modeled as the sum of K weighted underlying distributions, each representing the distribution of a gene conditional on membership in each cluster. The entire data likelihood is then a product across all the genes. Once again, this approach does not specify any correlation between genes in the same cluster. These types of mixture models are valid for clustering patients, but do not reasonably extend to the setting of clustering features measured on each patient.

A statistically sound approach for model-based clustering is to model the genes by including a random effect such that highly correlated genes fall in the same cluster. The paper [10] implemented an EM algorithm to fit a random effects model for clustering. Alternatively, the Bayesian paradigm provides a unified framework for fitting complex hierarchical models. For example, [11] proposed a random effects clustering model and performed a stochastic search for clusters using the posterior distribution of the unknown partition as the objective function. A Markov chain Monte Carlo (MCMC) sampling scheme for simultaneously selecting discriminating genes and clustering patients is presented in [12]. The Dirichlet process prior has been developed as a Bayesian prior for estimating normal means and can be used as a prior for regression parameters in the clustering setting as seen in [13].

A wide range of methods have been developed for finding meaningful gene clusters that are associated with a response or outcome [14–17]. Recently, [18] proposed a method that adds a penalty to the regression likelihood to enforce equal regression coefficients within the same cluster. Despite widespread interest in this topic, there is still much room for improvement [19]. In this article, we describe a fully model-based approach that is easily extendable to more complicated data structures. We propose a joint model for simultaneously clustering correlated genes and predicting a continuous patient outcome. We use a random effects model to describe gene expression cluster membership and relate the latent cluster effects to a continuous patient outcome via a linear model. We develop a MCMC clustering algorithm based on a marginalized likelihood for model fitting and parameter inference. The Bayesian framework allows the outcome to drive the clustering of genes when fitting the joint model and accounts for variation around the cluster membership parameter. By simultaneously modeling patient outcome with gene expression and developing a clustering algorithm that makes use of clinical data, we can potentially generate clusters that are more useful for describing the clinical course of injury.

## Methods

### Model Specification

We propose a joint model for simultaneously clustering correlated gene expression data and predicting a continuous patient outcome. Consider representing the microarray dataset as a *N* × *J* matrix consisting of gene expression values for *J* genes measured on *N* patients. Let *Y*_*ij*_ be the gene expression value for patient *i* and gene *j* belonging in cluster *k*. Conditional on membership of gene *j* in the *k* ^*th*^ cluster, the random effects model for describing gene expression is

$$Y_{ij}=c_{ik(j)}+\epsilon_{ij}$$ (1)

where *i* = 1, … ,*N*, *j* = 1, …, *J*, and *k* = 1, …, *K*. Here, *c*_*ik*(*j*)_ are patient-cluster specific random effects that represent the cluster centers and induce correlation between genes in the same cluster. We assume *c*_*ik*(*j*)_ ∼ *N*(0, *τ* ^2^) after the data have been log-transformed and centered to have mean zero. This can be accomplished by subtracting the *J* column means. Thus, for a given patient, the covariance between genes in the same cluster is *τ* ^2^, while genes in different clusters and across different patients remain independent. The *ϵ*_*ij*_ are measurement errors, assumed to be distributed *N*(0, *σ* ^2^). To link the gene clusters to patient outcome, we specify a linear relationship between the clusters and *Z*_*i*_, where *Z*_*i*_ is a continuous outcome for patient *i*,

$$Z_{i}=\sum_{k=1}^{K}\beta_{k}c_{ik(j)}+\xi_{i}.$$ (2)

The cluster effects *c*_*ik*(*j*)_ relate gene expression and patient outcome to each other by acting as covariates in the regression model. The *β*_*k*_ are the respective regression coefficients for each cluster, and the error terms are assumed to be *ξ*_*i*_ ∼ *N*(0, *γ* ^2^).

### Likelihood

The patient-cluster specific random effects, *c*_*ik*(*j*)_, are introduced as a convenience in describing our model, but since the *Y*_*ij*_ and *Z*_*i*_ are multivariate normal, we can use the likelihood function after marginalizing out the random effects. In fact, let *X*_*i*_ = (*Y*_*i*_, *Z*_*i*_) be the vector of observations on gene expression and outcome associated with patient *i*, where *Y*_*i*_ = (*Y*_*i*1_, …, *Y*_*iJ*_). Let *ϕ* = (*ϕ*_11_, …, *ϕ*_1*K*_, *ϕ*_21_, …, *ϕ*_*JK*_), where *ϕ*_*jk*_ is an indicator denoting membership of gene *j* in cluster *k* and let Θ denote the set of parameters {*σ*, *τ*, *β*, *γ*, *ϕ*}. The resulting likelihood for (*Y*, *Z*) is given by a multivariate normal distribution,

$$f\left(Y,Z|\Theta \right)=\prod_{i=1}^{N}\frac{exp\{-\frac{1}{2}X_{i}^{\prime }\Sigma^{-1}X_{i}\}}{{\left(2\pi \right)}^{(J+1)/2}{\left|\Sigma \right|}^{1/2}}.$$ (3)

The covariance matrix *Σ* is a symmetric (*J* + 1) × (*J* + 1) matrix and if we reorder the *Y*_*ij*_ so the genes in a common cluster are adjacent, then it is block diagonal in all but the last row and column. If we let *u* = (1, …, *J*) and *v* = (1, …, *J*) index the matrix elements, and let *S*_*k*_ denote the *k* ^*th*^ cluster set, then the covariance matrix is represented by

$$\begin{array}{c} \Sigma_{u,v}=\sigma^{2}I\left(u=v\right)+\tau^{2}\sum_{k=1}^{K}I\left(u,v\in S_{k}\right)\\ \Sigma_{u,J+1}=\tau^{2}\sum_{k=1}^{K}I\left(u\in S_{k}\right)\beta_{k}\\ \Sigma_{J+1,J+1}=\tau^{2}\sum_{k=1}^{K}\beta_{k}^{2}+\gamma^{2} \end{array}$$ (4)

Note that the last equation is not completely identifiable if a cluster, say *k*, is empty. In that case, $\beta_{k}^{2}\tau^{2}+\gamma^{2}$ can take the same value for different values of *β*_*k*_ and *γ* ^2^. However, neither the value of *β*_*k*_ for the empty clusters nor *γ* ^2^ is of interest, so this non-identifiability does not effect the estimation of the parameters of interest.

Finding the maximum likelihood estimates of the parameters of this model is not possible because each choice of clusters for the set of genes is a distinct set of parameter values. To examine all of these would require too many evaluations of the likelihood. Rather than attempting this, we assume a prior distribution for the parameters in order to get a probability distribution of the parameter values. If we generate a large sample from this distribution than the distribution of this sample will approximate the sampling distribution for the parameters. We use a Gibbs sampler to generate this sample. This takes advantage of the fact that the conditional distribution of each parameter given the other parameters is relatively simple. By looping through the parameters in a specified order and in each case generating the next parameter from the conditional distributions given the previous ones we generate a sequence of parameter values which will eventually have the distribution described above. The algorithm we used is among the class of algorithms called Markov Chain Monte Carlo (MCMC). The use of an MCMC is computationally feasible because a closed form expression for both the inverse and the determinant of Σ exists. Therefore, the expression for the multivariate normal distribution simplifies substantially. In addition, speed-up can be accomplished by updating the log likelihood when gene *j* is moved from one cluster to another rather than recomputing it.

### Prior distributions

We specify a non-informative prior distribution for every parameter. We assume that *σ*, *τ* and *γ* have a uniform prior distribution from 0 to *A*, where *A* is large [20]. We assume the regression parameters *β* are independent with uniform priors on a wide interval of the real line centered at 0.

Let *ω*_1_, …, *ω*_*K*_ be the proportion of genes in each cluster. Non-informative conjugate priors are specified for *ω* and *ϕ*. A symmetric Dirichlet prior is set for the weights, *P*(*ω*_1_, …, *ω*_*K*_) ∝ Dirichlet(*α*, …, *α*). Larger values of *α* reflect the presence of more non-empty clusters while smaller values of *α* reflect fewer non-empty clusters. Lastly, the cluster membership variable *ϕ* has a multinomial prior that depends on the weights, *P*(*ϕ*_*jk*_ = 1) = *ω*_*k*_.

### Model Fitting

We fit the model by implementing a MCMC algorithm that consecutively samples every parameter until a sufficient representation of the posterior distribution is achieved. When the conditional distribution of a parameter cannot be directly sampled from, we use the Metropolis-Hastings algorithm. Candidate values are drawn from a proposal distribution and accepted with probability proportional to the ratio of the likelihood evaluated at the new value to the likelihood evaluated at the old value, divided by the ratio of the probability of the new value in the proposal distribution evaluated at the old value to the old value in the proposal distribution evaluated at the new value. That is, if *Q* is the proposal density, *P* is the posterior likelihood, *θ*′ is the current parameter value, and θ* is the candidate parameter value, then samples are accepted with probability

$$min(1,\frac{P\left(\theta *|Y,Z\right)/Q\left(\theta *|\theta^{\prime }\right)}{P\left(\theta^{\prime }|Y,Z\right)/Q\left(\theta^{\prime }|\theta *\right)})$$ (5)

The MCMC sampling procedure consists of repeating the following four steps until convergence:

1. Sample each of *σ* ^2^, *τ* ^2^, and *γ* ^2^ using a Metropolis-Hastings algorithm with an inverse gamma proposal distribution with shape parameter *s* and scale parameter *x*/*θ*, where *x* is the inverse of the previous value of the parameter.
2. For each gene, do the following two steps
  - Calculate the likelihood of the gene belonging in each of the *K* clusters using (3). The value of the likelihood weighted by the current value of *ω* then becomes the updated multinomial sampling probabilities. Then this multinomial is sampled and the gene is placed in the designated cluster.
  - The regression coefficients are sampled whenever there is a change in the cluster membership of a gene and all *K* are sampled again when all the genes have been reassigned.
3. Once all the genes have been considered for movement, sample the weights corresponding to the chance of a gene being in each cluster, *ω*, using a Dirichlet(*α* + *n*_1_, …, *α* + *n*_*K*_) distribution, where *n*_*k*_ is the current number of genes in the *k* ^*th*^ cluster.

### Number of clusters *K*

So far we have been using *K* to denote the number of clusters, but to be precise, we should in fact think of *K* as the maximum number of possible clusters. *K* is a fixed value and is not sampled in the algorithm. The idea is that the genes do not necessarily need to group into exactly *K* clusters. In other words, one can think of *K* as the number of filled and empty clusters. To understand how this can happen, recall that *P*(*ϕ*_*jk*_ = 1) for all *j*, *k* is proportional to the weighted likelihood of belonging in cluster *k*. These multinomial probabilities are always non-zero because *ω*_*k*_ is positive for all *k*, regardless of cluster size. As a result there is always a chance that a cluster will end up with no genes, or that an empty cluster will become filled at any given iteration due to the probabilistic nature of the allocation. As the genes become grouped and regrouped, a subset of the *K* clusters becomes filled. The subset of filled clusters is what we naturally think of as the number of clusters. This value changes at very iteration and follows as an immediate result of the cluster membership parameter.

### Starting Values and Number of Chains

One issue in the use of the MCMC is whether the chain traverses all the clusterings that have a high likelihood. What we suggest is to run two chains (each starting with different random assignment of genes to clusters) and calculate the concordance of the two chains. We define the concordance of gene *i* with gene *j* as the proportion of times they are in the same cluster. If one plots the degree of concordance between every two genes in one chain versus the other, it will be apparent whether the two chains ended in the same place. If they didn’t, one should run say, ten chains and look at the first five verses the last five. If the plot shows good correspondence, then five chains are adequate to transverse the space. These numbers are just rough guidelines. It is possible to develop statistical rules for this based on a model for the number of possible distinct clusterings.

### Describing Results

As expected, the MCMC algorithm will not ordinarily reduce to a single set of clusters for two reasons. First, unless *τ* ^2^ is very large compared to *σ* ^2^, or there is a very large sample size, the sampling distribution of cluster membership will contain several different possible clusterings. Secondly, there could be label switching, whereby the genes that are grouped together are the same for two runs, but the cluster identities can be switched. The other cluster-dependent parameters, *β* and *ω*, can also appear non-identifiable as a consequence of label switching. Consider the case of three genes with *K* = 3. Suppose at one iteration genes 1 and 2 are in cluster A, gene 3 is in cluster B, and cluster C is empty. Suppose that in the first step of the clustering iteration, gene 1 and gene 2 are switched to cluster C and gene 3 is left alone in cluster B. Now we have a case of label switching.

Given these problems, the way we describe the results is to use a concordance heat map. This is a graph with genes on each of the axes and the probability of genes *i* and *j* being in the same cluster represented by the intensity and color of the point at (*i*, *j*). The genes are ordered by putting genes with high concordance next to each other. Displaying gene concordance on a heat map allows the relationship between genes to be captured and circumvents the issue of label switching which can cause the appearance of non-identifiability. Looking at the map can tell which genes tend to cluster together.

In addition to displaying the gene clustering, we would also like to use a patient’s microarray data to predict their outcome. For this we note that the predictive density of that patient’s outcome can be obtained. Since *Z*_*i*_ and *Y*_*i*_ are both normally distributed, *f*(*Z*_*i*_|*Y*_*i*_) is also normally distributed. Its expected value and variance are given by

$$E\left(Z_{i}|Y_{i}\right)=\tau^{2}\sum_{k=1}^{K}\frac{\beta_{k}}{\sigma^{2}+n_{k}\tau^{2}}\left(\sum_{j\in S_{k}}Y_{ij}\right)$$ (6)

$$Var\left(Z_{i}|Y_{i}\right)=\tau^{2}\sigma^{2}\sum_{k=1}^{K}\frac{\beta_{k}^{2}}{\sigma^{2}+n_{k}\tau^{2}}+\gamma^{2}.$$ (7)

## Simulations

Simulations were conducted to evaluate the performance of our algorithm and to study the effect of outcome inclusion and different parameter values on the resulting clusters. Data consisted of 80 patients and 50 genes arising from 3 clusters. We considered various values of *τ* to assess the ability of our method to detect the correct cluster structure when cluster variation is low and when cluster variation is high compared to the variation in the residual error. This ratio, *τ* ^2^/*σ* ^2^, is what we will refer to as the variance ratio. The remaining parameter values were set to *σ* = 1, *γ* = 1, *β*_1_ = −5, *β*_2_ = 0, and *β*_3_ = 5. We set *α* = 1 for the Dirichlet prior and *K* = 5. In our simulations, one did not need multiple chains. For every dataset, we ran 1,000 iterations and discarded 500 as burn-in.

A visual representation of the simulation results can be depicted as a heat map that shows the proportion of iterations that every pair of genes is assigned to the same cluster. In the event of label switching, summarizing the output as a heat map aids in visualizing the groups, but even in the absence of label switching, the heat map has the advantage of providing information about the uncertainty surrounding the allocations. We do not assume that *ϕ* is a fixed value, but rather a parameter with a distribution where some groupings are more likely than others. The genes are listed along both axes in the same order, grouped together by their true cluster membership. Concordance is represented as a gradient from white (0%) to black (100%) with 16 discrete shades of gray.

**Fig 1:**
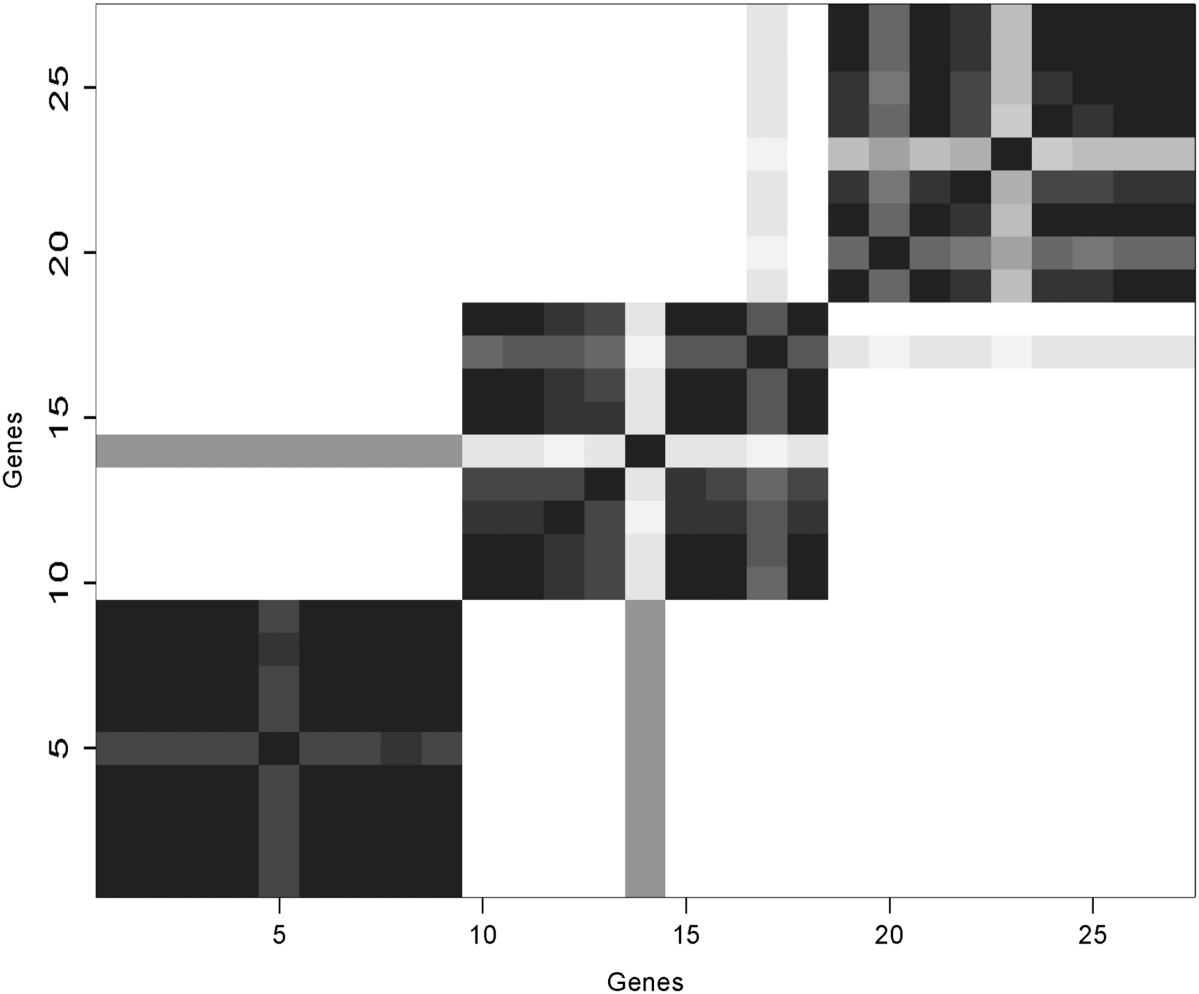
Concordance Maps for Simulated Data with Outcome Included. Concordance varies from 0% (white) to 100% (black). When the variance ratio (*τ* ^2^/*σ* ^2^) is small, including outcome produces clearer clusters.

**Fig 2:**
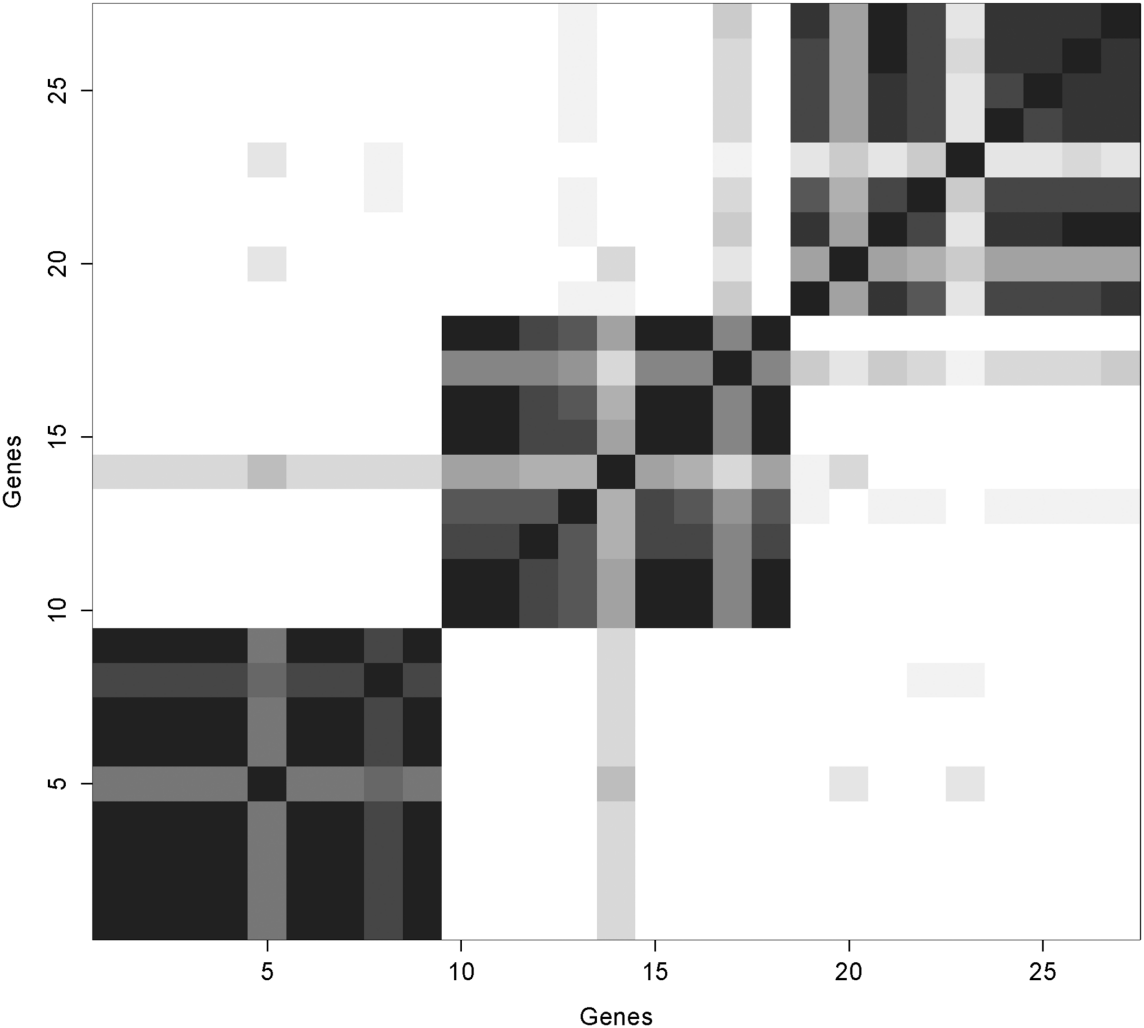
Concordance Maps for Simulated Data with Outcome Excluded. Concordance varies from 0% (white) to 100% (black). When the variance ratio (*τ* ^2^/*σ* ^2^) is small, including outcome produces clearer clusters.

When the variance ratio *τ* ^2^/*σ* ^2^ is low (.15), there is more variability in the clustering and the clustering is more determined by outcome. This effect is modest, as depicted in the heat maps for the models with and without outcome shown in Figs 1 and 2. When the variance ratio is high (we used a value of 4), all the chains converge to the same clustering and the clustering of each gene is completely stable. Furthermore, in this case, the use of the outcome does not improve the clustering. The parameter estimates from the simulations are shown in Table 1. When there were only three clusters of 9 genes each, the proportion of correctly paired genes was.82 when outcome was not included and moved to.88 with outcome included. When the number of clusters increased, the difference in these proportions diminished to only .71 versus .66.

**Table 1:** Simulation Results: Parameter estimates resulting from simulation for the model with and without outcome with N = 80 patients, J = 50 genes, and 100 replications with 1000 iterations each.

Parameter	True value	Mean	SE	Mean of SE
*σ*	1	0.99	0.03	0.03
*τ*	4	4.09	0.38	0.35
*β*_1_	-5	-4.99	0.12	0.13
*β*_2_	0	-0.03	0.13	0.13
*β*_3_	5	4.97	0.14	0.14

## Trauma Data Analysis

We applied our methodology to the Inflammation and Host Response to Injury trauma dataset, a rich dataset that contains information on numerous factors related to the biology of inflammation following severe traumatic injury. There are a total of 167 patients in the trauma dataset, each of whom has their blood leukocyte expression levels measured on an Affymetrix microarray chip consisting of 54,674 probe sets (which we will henceforth call ‘genes’). The full dataset consists of microarrays that have been taken at seven different time points following the patients’ injury, starting from immediately after the injury to up to 28 days later. For our analysis however, we restrict ourselves only to microarray data collected on day four from the 147 patients who are still in the intensive-care unit at that time.

The gene expression values have been pre-processed using dChip, log-transformed and centered prior to analysis. We use a subset of 87 genes for our cluster analysis. These genes were pre-selected by Glue investigators to be those that had significant differential expression with at least a two-fold difference between patients with complicated versus uncomplicated recovery. Our objective is to find clusters of genes that are associated with each other as well as associated with a relevant patient outcome. The outcome that we use in our analysis is maximum multiple organ failure (MOF), a continuous score that describes the severity of the patient’s multiple organ failure and is predictive of metabolic recovery. MOF is the cumulative sum of individual scores from the respiratory, renal, hepatic, cardiovascular, and hematologic components, each ranging in value from 0 to 4 for least to most severe. The resulting groups of genes can then be examined for their functional relationships and interdependent roles in the inflammation response pathway.

We ran two MCMC chains, each starting from a different set of randomly chosen over-dispersed starting values. Non-informative priors were specified for all the parameters, and hyper-parameters were chosen to be *α* = 1 and *K* = 20. Since both chains yielded the same clustering, we only used one chain. We ran 3,000 iterations with 2,000 discarded as burn-in.

**Fig 3:**
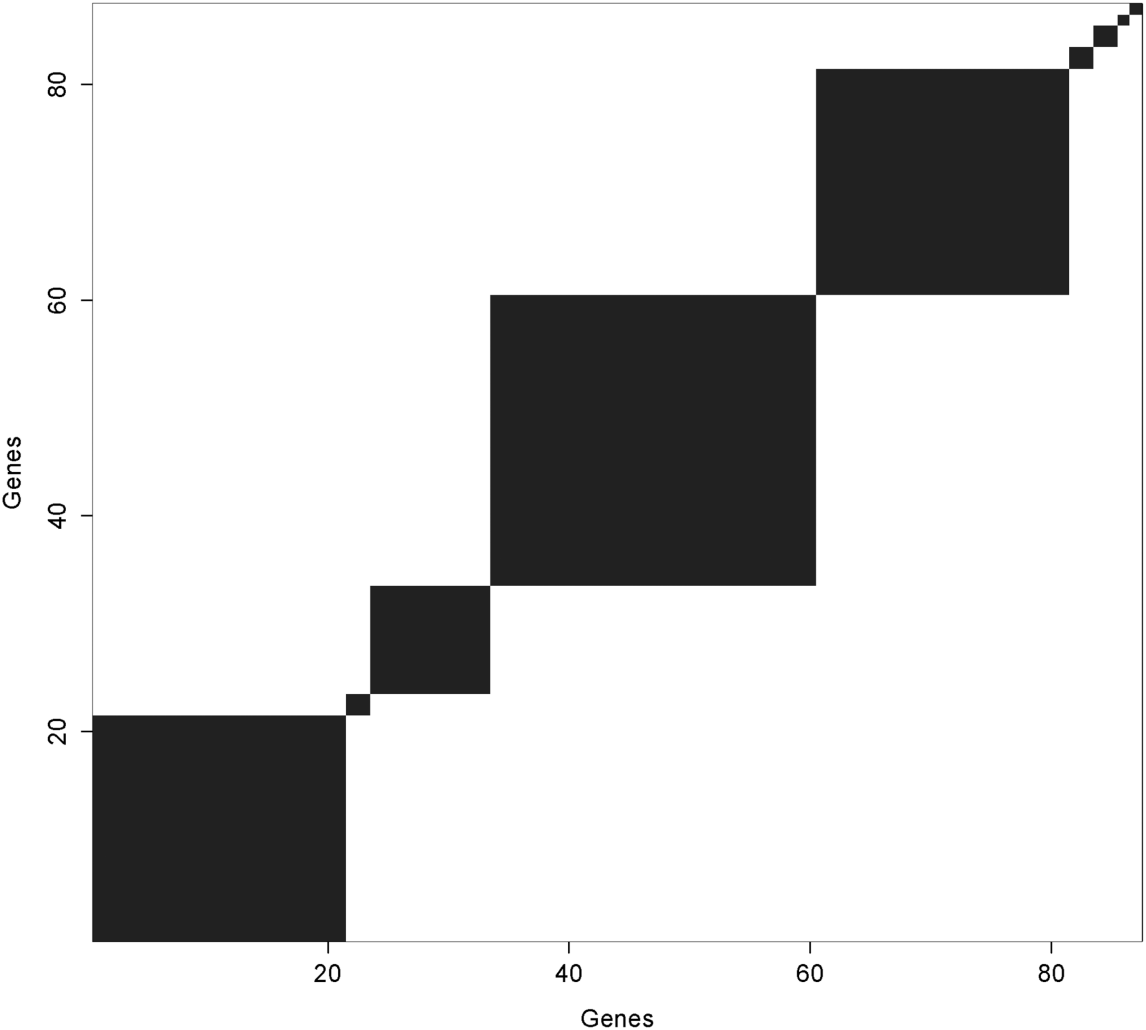
Concordance Map For Glue Trauma Data with Outcome Included.

The mean of the variance ratio is near 4 both with and without outcome. From simulations we know that this predicts good clustering whether or not outcome is included as the uncertainty surrounding cluster membership is minimal because the estimated variance ratio is relatively large. The coefficient estimates are conditional on *K* = 10, where the clusters range from size 1 to size 25. Only those iterations for which the genes in each respective cluster exclusively group together are used in calculating the coefficient estimate for that cluster. Fig 3 shows the concordance map for the Glue Grant trauma data when the outcome was included. We sorted the genes (as noted earlier) so that the genes in the same clusters are adjacent. All but two of the clusters were stable across all the iterations. There were two clusters which included only two genes that moved about. The genes are listed in order by decreasing values of *β* in Table 2.

**Table 2:** List of Genes Sorted by Increasing *β*.

*β*	Gene Symbol	Gene Name
-8.544	MMP8	Matrix metallopeptidase 8 (neutrophil collagenase)
-8.544	MMP8	Matrix metallopeptidase 8 (neutrophil collagenase)
-8.544	OLFM4	Olfactomedin 4
-8.544	LCN2	Lipocalin 2
-8.544	CD24	CD24 molecule
-8.544	LTF	Lactotransferrin
-8.544	CEACAM8	Carcinoembryonic antigen-related cell adhesion molecule 8
-8.544	TCN1	Transcobalamin I (vitamin B12 binding protein, R binder family)
-8.544	CD24	CD24 molecule
-8.544	CEACAM6	Carcinoembryonic antigen-related cell adhesion molecule 6
-3.047	PTGS2	Prostaglandin-endoperoxide synthase 2 (prostaglandin G/H synthase and cyclooxygenase)
-3.047	PTGS2	Prostaglandin-endoperoxide synthase 2 (prostaglandin G/H synthase and cyclooxygenase)
-0.219	HLA-DQB1	Major histocompatibility complex, class II, DQ beta 1
-0.219	HLA-DQA1	Major histocompatibility complex, class II, DQ alpha 1
2.155	OLAH	Oleoyl-ACP hydrolase
2.155	OLAH	Oleoyl-ACP hydrolase
2.222	IFIT2	Interferon-induced protein with tetratricopeptide repeats 2
2.222	HLA-DRA	Major histocompatibility complex, class II, DR alpha
2.222	HLA-DPA1	Major histocompatibility complex, class II, DP alpha 1
2.222	EPSTI1	Epithelial stromal interaction 1 (breast)
2.222	HLA-DRA	Major histocompatibility complex, class II, DR alpha
2.222	HLA-DRB1	Major histocompatibility complex, class II, DR beta 1
2.222	OAS3	2’-5’-Oligoadenylate synthetase 3, 100kDa
2.222	HLA-DRB1	Major histocompatibility complex, class II, DR beta 1
2.222	HLA-DRB1	Major histocompatibility complex, class II, DR beta 1
2.222	HLA-DPB1	Major histocompatibility complex, class II, DP beta 1
2.222	PMAIP1	Phorbol-12-myristate-13-acetate-induced protein 1
2.222	HLA-DMB	Major histocompatibility complex, class II, DM beta
2.222	HLA-DMA	Major histocompatibility complex, class II, DM alpha
2.222	HLA-DRB1	Major histocompatibility complex, class II, DR beta 1
2.222	GNLY	Granulysin
2.222	TGFBI	Transforming growth factor, beta-induced, 68kDa
2.222	HLA-DPA1	Major histocompatibility complex, class II, DP alpha 1
2.222	GNLY	Granulysin
2.222	OAS1	2’-5’-oligoadenylate synthetase 1, 40/46kDa
2.222	CD74	CD74 molecule, major histocompatibility complex, class II invariant chain
2.222	HLA-DQB1	Major histocompatibility complex, class II, DQ beta 1
2.701	TDRD9	Tudor domain containing 9
2.701	ANKRD55	Ankyrin repeat domain 55
2.701	VNN1	Vanin 1
2.701	NSUN7	NOP2/Sun domain family, member 7
2.701	VNN1	Vanin 1
2.701	CDK5RAP2	CDK5 Regulatory subunit associated protein 2
2.701	OLAH	Oleoyl-ACP hydrolase
2.701	CDK5RAP2	CDK5 regulatory subunit associated protein 2
2.701	IL1R2	Interleukin 1 receptor, type II
2.701	LOC399972	Hs.585206 hypothetical LOC399972
2.701	MIAT	Myocardial infarction associated transcript (non-protein coding)
2.701	IL1R2	Interleukin 1 receptor, type II
2.701	ENSG000002542	Homo sapiens, clone IMAGE:4696931, mRNA
2.701	NAIP	NLR family, apoptosis inhibitory protein
2.701	SLC26A8	Solute carrier family 26, member 8
2.701	LRG1	Leucine-rich alpha-2-glycoprotein 1
2.701	GRB10	Growth factor receptor-bound protein 10
2.701	ATP6V1C1	ATPase, H+ transporting, lysosomal 42kDa, V1 subunit C1
2.701	IL1R1	Interleukin 1 receptor, type I
2.701	HGF	Hepatocyte growth factor (hepapoietin A; scatter factor)
2.701	PDGFC	Platelet derived growth factor C
2.701	GALNT14	UDP-N-acetyl-alpha-D-galactosamine:polypeptide N-acetylgalactosaminyltransferase 14
2.701	DACH1	Dachshund homolog 1 (Drosophila)
2.701	AGFG1	ArfGAP with FG repeats 1
2.701	LOC100127983	Hypothetical protein LOC642730
2.701	FOLR3	Folate receptor 3 (gamma)
2.701	SIPA1L2	Signal-induced proliferation-associated 1 like 2
3.559	IFIT1	Interferon-induced protein with tetratricopeptide repeats 1
3.559	IFI44L	Interferon-induced protein 44-like
3.559	RSAD2	Radical S-adenosyl methionine domain containing 2
3.559	RSAD2	Radical S-adenosyl methionine domain containing 2
3.559	IFI44	Interferon-induced protein 44
3.559	HERC5	Hect domain and RLD 5
3.559	IFIT3	Interferon-induced protein with tetratricopeptide repeats 3
3.559	IFIT3	Interferon-induced protein with tetratricopeptide repeats 3
3.559	EPSTI1	Epithelial stromal interaction 1 (breast)
3.559	IFIT2	Interferon-induced protein with tetratricopeptide repeats 2
3.559	CMPK2	Cytidine monophosphate (UMP-CMP) kinase 2, mitochondrial
3.559	MX1	Myxovirus (influenza virus) resistance 1, interferon-inducible protein p78
3.559	IFI6	Interferon, alpha-inducible protein 6
3.559	XAF1	XIAP associated factor 1
3.559	ISG15	ISG15 ubiquitin-like modifier
3.559	IFI44	Hs.82316 Interferon-induced protein 44
3.559	XAF1	XIAP associated factor 1
3.559	IFIT5	Interferon-induced protein with tetratricopeptide repeats 5
3.559	XAF1	XIAP associated factor 1
3.559	OAS2	2’-5’-oligoadenylate synthetase 2, 69/71kDa
3.559	IFIT5	Interferon-induced protein with tetratricopeptide repeats 5
N/A	PCOLCE2	Procollagen C-endopeptidase enhancer 2
N/A	THBS1	Thrombospondin 1

## Discussion

We have proposed Bayesian methodology for the informative clustering of genes. Our model accounts for correlation between genes in the same cluster and jointly relates the gene expression values to a continuous patient outcome such that this additional information helps drive the clustering of the genes.

In this paper we focus on how this method can be used as a clustering algorithm and not on whether the predictions it provides are better than other machine learning algorithms. We have this focus because the best machine learning algorithm depends on the unknown underlying structure of the data. Assuming a model allows us to find estimates for the relationship between the gene clusters and the outcome which can inform an understanding of which genes act to increase versus decrease the outcome measure.

It may be worthwhile to consider relaxing some of the assumptions of our model. The proposed model is based on the assumption that there is a true underlying clustering and genes in different clusters are independent. Additionally, the model assumes that groups of correlated genes that provide the same information have the same effect on outcome. Two genes that are marginally independent and have the same effect on outcome will go into two different clusters because genes in the same group would be correlated. More problematic are genes that are correlated but have different relationships with outcome. If they were put in separate clusters, then the clusters would not be independent. This could occur if the deviance of a gene about the random cluster effect was itself prognostic. One could expand the model to allow different effects in the genes of a cluster, but in that case it might be better to cluster on association between genes and then separately calculate the regression coefficients.

Another variation to the model that allows for more flexibility includes allowing a heterogeneous covariance structure where a different *τ*_*k*_ is specified for every cluster. A non-linear relationship between the clusters and outcome could also be modeled. It would be worthwhile to consider incorporating global moves in the algorithm such as splitting or combining clusters. Though this would allow the partition space to be explored more fully, it would add extra computational complexity.

Our model can be extended to accommodate categorical outcomes using a probit or logistic model, or time to event outcomes using semi-parametric models. Additionally, the model can be extended to the longitudinal microarray setting where it is assumed that groups of genes cluster together in their patterns over time.

Uncovering the underlying cluster structure of gene expression data and determining the functional properties of the gene clusters will help us understand the biological basis of events following traumatic injury. Developing a reliable method of predicting patient recovery can save valuable resources that are required for careful monitoring of every patient. If we can successfully accomplish these objectives, we can develop intervention strategies that have the potential of putting more patients on the road to recovery.

The programs used for this paper (both for simulations and data analysis) and the Glue Grant are available in the *R* archive under the name “supcluster”.
